# Investigation of Y/SBA Composite Molecular Sieves Morphology Control and Catalytic Performance for *n*-Pentane Aromatization

**DOI:** 10.1038/srep23826

**Published:** 2016-03-31

**Authors:** Chun-Wei Shi, Wen-Yuan Wu, Shuai Li, Xue Bian, Shan-lin Zhao, Ming-Yuan Pei

**Affiliations:** 1 College of Chemistry, Chemical Engineering and Environmental Engineering, Liaoning Shihua University, Funshun Liaoning 113001, China; 2 School of Materials and Metallurgy, Northeastern University, Shenyang Liaoning 110819, China; 3 Institute of Pharmaceutical Sciences of Shandong Province, Key Laboratory for Chemical Drug Research of Shangdong Province, Jinan Shandong 250101, China; 4 Inha University, 100 Inharo, Nam-gu Incheon 402-751, Korea

## Abstract

Using Y molecular sieve as the core, Y/SBA-15 composite molecular sieves were prepared by different crystallization methods in the paper. The growth process and morphologies of the composite molecular sieves were controlled by adjusting the synthesis factors. The structures and acidity of two kinds of composite molecular sieves were characterized by X-ray diffraction (XRD), N_2_ adsorption/desorption, transmission electron microscopy (TEM), and NH_3_-TPD. The catalysis performances of the composite molecular sieves were investigated in the aromatization reaction of *n*-pentane. The results indicated that the desired core-shell composite molecular sieves were obtained when the crystallization conditions were 36 hours, 100 °C and secondary crystallization. The aromatization results showed that core-shell composite molecular sieves had better selectivity for producing high application value xylenes compared to mixed-crystal composite molecular sieves.

Microporous molecular sieves have relatively strong acidity^1^,^2^,^3^. However, their open-frameworks and relatively small pore sizes limit their catalytic effects on the reactions of macromolecules. Compared to microporous molecular sieves, mesoporous molecular sieves^4^ have larger pore sizes, specific surface area and pore wall thickness. However, they also have several disadvantages such as insufficient active sites, amorphous pore walls and poor hydrothermal stability of the mesoporous pure silica material.

Composite molecular sieves are increasingly popular, as they help combine the advantages of different types of molecular sieves while limiting their drawbacks. Among the different composite molecular sieves with hierarchical porous structures, composite molecular sieves with core-shell structure have shown outstanding catalytic performance^5^,^6^,^7^. Recently, Ana *et al.*^8^ prepared HZSM-5/Al-MCM-41 core-shell composite molecular sieves using a dual-template method. Core-shell composite molecular sieves have inner and outer channels with different pore sizes (micro- and meso-porous) as well as appropriate acidity. The advantages of microporous and mesoporous molecular sieves are complimentary to each other, which can significantly improve the catalytic performance of composite molecular sieves^9^.

The demand for light aromatics BTX (benzene, toluene and xylenes) as important organic chemical raw materials and free blending components has increased progressively^10^. Recently, transformation of light alkanes into aromatics with high added value has attracted considerable attention. The aromatization process is accompanied by side reactions such as pyrolysis and coking^11^, and thus requires careful selection of catalysts. In recent years, molecular sieves such as HZSM-5^12^, ZSM^13^, and Y^14^ have been widely used to design catalysts for aromatization of light alkanes. Compared to mono-molecular sieves, composite molecular sieves (Hβ/HZSM-5, HZSM-5/AlMCM-41, etc.)^15^,^16^ possess more desirable properties which favor the aromatization reaction. Hai Wan *et al.*^17^ obtained ZSM-5/LK composite molecular sieves catalysts via different crystallization methods to investigate their performance in aromatization of light alkanes and found that molecular sieves prepared via secondary crystallization showed better aromatization catalysis performance.

In this study, we synthesized a composite molecular sieve with SBA-15 as shell and Y as core by using a hydrothermal method. The effects of the main crystallization conditions, including time and temperature, on the open-framework structure and acidity of the resulting composite molecular sieves were investigated. The catalytic performances of the prepared molecular sieves were evaluated in the *n*-pentane aromatization reaction by monitoring the product yields.

## Experimental

Sodium aluminate, sodium hydroxide, sodium silicate, cerium nitrate, heptanes, toluene and tetraethyl orthosilicate (TEOS) were all analytical pure reagents and purchased from Sinopharm Group Co. LTD. Triblock copolymer poly (ethylene glycol)-block-poly (propylene glycol)-block-poly (ethylene glycol) (Pluronic P123) was purchased from Sigma Aldrich, America. Y type microporous materials is provided by Fushun Petrochemical Research Institute.

### Composite molecular sieves mother liquors

To a three-necked flask, triblock copolymer P123, H_2_O, TEOS, and HY were added in the weight ratio *m*(P123):*m*(H_2_O):*m*(TEOS):*m*(HY) of 4:30:9.5:4. Then, hydrochloric acid of a certain concentration was slowly added into the mixture to adjust the pH to 2–3. The mixture was agitated for a certain amount of time and then placed into the hydrothermal reactor. The solutions were crystallized in the oven at 100 °C for 10, 24, 36, and 48 hours.

### Sample by single crystallization

The mother liquor (30 ml) was placed in the oven at 100 °C keeping 36 hours for crystallization. After completion of the reaction, the product was filtered, dried and incinerated to give the single crystallized Y/SBA-15 micro-/meso-porous composite molecular sieve, labeled as YS-S.

### Sample by secondary crystallization

The mother liquor (30 ml) was placed in the 60 °C oven for 10 hours and followed another 26 hours in the 100 °C oven for crystallization. When the reaction was completed, the product was dried and incinerated to give the Y/SBA-15 micro-/meso-porous composite molecular sieve of secondary crystallization, labeled as YS-D.

### Catalyst characterization

XRD patterns of the catalysts were obtained with a XRD-700 X-ray diffractometer in the 2 theta ranges of 0.5–70° (Rigaku Co.) by using Cu Ka radiation combined with a Ni filter. The N_2_ adsorption-desorption isotherms were measured at −196 °C with a Auto Chemisty 2010 instrument. Transmission electron microscopy (TEM) micrographs were collected on a Philips CM200 microscope. NH_3_-TPD patterns were recorded on a RC-IR-TP-3030 (Science and Technology Development Co. Ltd., China) to detect surface acidity of the molecular sieves. TPD of NH_3_ experiments were performed using 100 mg samples. After pretreatment in He environment at 400 °C for 1 h, samples were cooled to room temperature and then saturated with anhydrous NH_3_ (4% in He) at a flow rate of 30 ml/min for about 30 min. Desorption was carried out by heating the samples in He environment (40 ml/min) from 100 °C to 800 °C with a heating rate of 5 °C/min.

### Catalytic *n*-pentane aromatization reaction

A MRT-6103 10 ml fixed bed micro-reactor, equipped with a stainless steel tube of 10 mm inner diameter was used. The calculation method of catalyst weight is as follows:


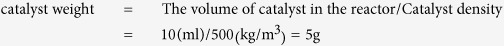


So the reactor was operated under atmospheric pressure with 5 g of catalyst YS-S and YS-D, respectively. The reaction conditions were as follows. The temperature was set to be 530 °C, the pressure was set to be 0.1 MPa, and the weight hourly space velocity (WHSV) was set to be 1 h^−1^. The product analysis was conducted on a SP-2000 gas chromatography (GC) instrument using 3 m of 8% organobentonite packed column. The temperature programming method was used. The column temperature was first held at 30 °C for 0.5 minutes, then raised to 80 °C at the rate of 2.5 °C/min, and held at 80 °C for 0.5 minutes. Afterwards, the temperature was raised to 160 °C at the rate of 5 °C/min and held at this temperature for 20 minutes. The inlet temperature was set to be 250 °C and the FID detector temperature was set to be 280 °C. The head pressure was 1000 KPa; the split ratio was 50:1; and the sample injection volume was 1 μL.

## Results and Discussions

### Effect of crystallization conditions on the composite molecular sieves-Crystallization time

**Figure 1:**
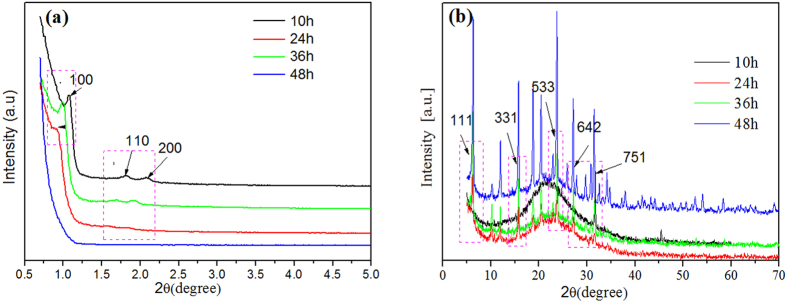
XRD spectra of Y/SBA-15 at different crystallization times (**a**) SWXD and (**b**) WXRD.

Figure 1 shows the XRD spectra of Y/SBA-15 composite molecular sieves prepared at 100 °C for different crystallization times. It can be seen from Fig. 1 that when the crystallization time was 10 hours, there was no mesoporous characteristic diffraction peak at 100, 110, or 200 in Fig. 1(a). Also, the intensities of the Y zeolite characteristic peaks at 111, 533, and 751 stayed constant in Fig. 1(b). Clearly, the desired reaction did not proceed at this short crystallization time, as the SBA-15 was still at the amorphous gel stage. When the crystallization time was extended to 24 hours, a weak mesoporous characteristic peak started to show up at 100 degrees. However, there was no characteristic peak at 110 or 200, indicating that formation of the mesoporous phase was incomplete. When the crystallization time was extended to 36 hours, the mesoporous characteristic peak gradually increased and the characteristic peak of SBA-15 appeared in the wide angle area along with the characteristic peak of Y, which indicated good degree of crystallization for both mesopores and micropores. When the crystallization time was extended to 48 hours, the characteristic peak intensities decreased slightly, perhaps indicating some degradation. Thus, the crystallization time of 36 hours is considered optimum for the SBA-15 to form a mesoporous phase with good crystallization degree on the surface of the microporous phase Y.

### Crystallization temperature

**Figure 2:**
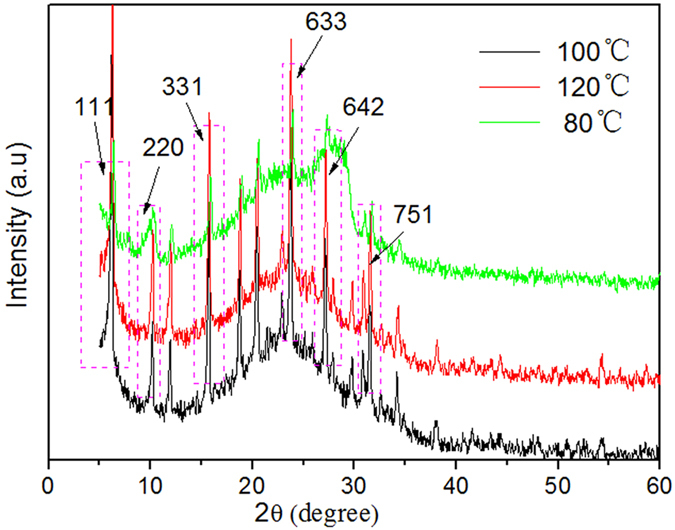
XRD spectra of composite molecular sieves prepared at different crystallization temperatures.

The crystallization temperature is known to have significant impact on the structure of the mesoporous molecular sieve SBA-15^13^. The investigated crystallization temperature range was set to be 80–120 °C and the crystallization time was set to be 36 hours. Figure 2 shows the XRD spectra of Y/SBA-15 samples prepared at different crystallization temperatures.

It can be seen from Fig. 2 that varying the crystallization temperature affects the intensities of the characteristic diffraction peaks of composite molecular sieves, indicating the open-framework of the Y/SBA-15 composite molecular sieves. As the crystallization temperature increased, the diffraction peaks shifted towards the small angle region and the interplanar spacing and the cell parameters increased, indicating that the pore size of the composite molecular sieves increased with increasing crystallization temperatures^5^. In addition, at higher crystallization temperatures, the intensities of the micro- and meso-porous characteristic diffraction peaks gradually increased, indicating that the temperature had a positive effect on improving the degree of order of the microporous and mesoporous phases. At low crystallization temperatures, there is insufficient energy provided for the reaction, leading to incomplete growth of mesoporous phase. On the other hand, high crystallization temperatures increase the production cost unnecessarily. Thus, a crystallization temperature of 100 °C was considered appropriate for our purposes.

### Single versus Secondary Crystallization

**Figure 3:**
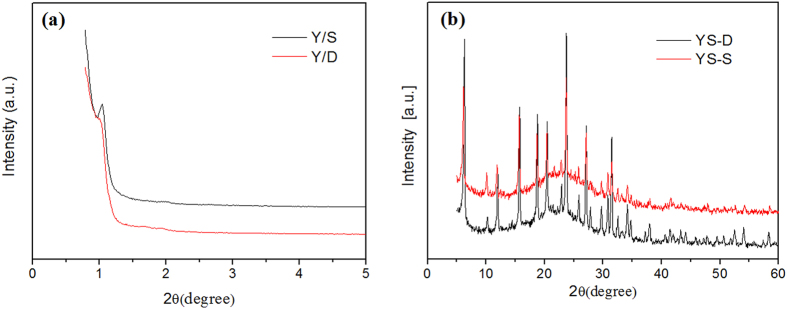
XRD spectra of YS-S and YS-D (**a**) SWXD and (**b**) WXRD.

The XRD spectra of samples YS-S and YS-D are shown in Fig. 3. Both the two samples show characteristic diffraction peaks of microporous Y and mesoporous SBA-15 as well as the highly ordered two dimensional hexagonal lattice structure.

**Figure 4:**
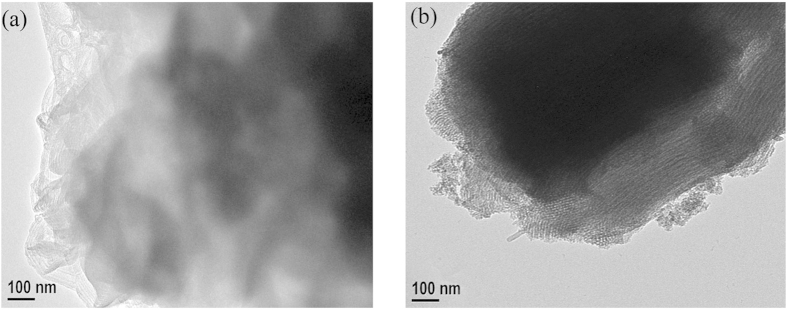
TEM images of Y/SBA-15 composite molecular sieves prepared via (**a**) single crystallization and (**b**) secondary crystallization.

The TEM images of YS-S and YS-D are shown in Fig. 4, and it can be seen that both of these two molecular sieves have microporous and mesoporous channels. It is evident from Fig. 4(a) that the composite molecular sieves prepared via single crystallization had issues of disordered crystal growth and irregular, floccus shaped channels. In Fig. 4(b), it can be seen that the composite molecular sieves prepared via secondary crystallization had relatively complete core-shell structure. The interface of the microporous and mesoporous phases was clearly visible and both of the channels were regular and ordered. In combination with the XRD characterization results in Fig. 3, it is confirmed that both YS-S and YS-D are composite molecular sieves.

Overall, the composite molecular sieves prepared by secondary crystallization had better morphology and crystal growth. This is due to the fact that the initial low temperature crystallization stage induced the process of nucleation, which prevented the disordered and irregular mesoporous channels formed by the splicing growth of the crystals due to the one-step high temperature crystallization. Secondary crystallization induced the ordered formation of mesoporous channels and had oriented synthesis effect on core-shell composite molecular sieves.

### Structural characteristics of the composite molecular sieves with different morphologies

**Figure 5:**
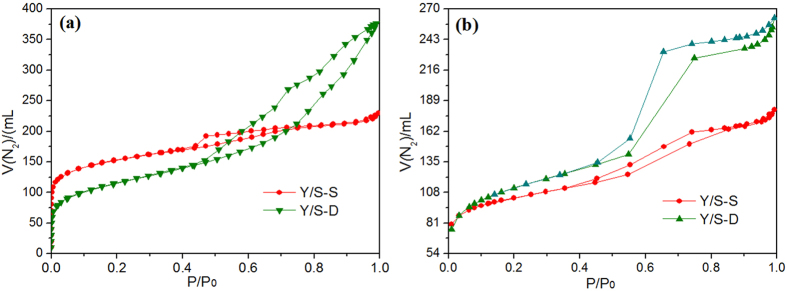
N_2_ adsorption-desorption isotherm of Y/S-S and Y/S-D. (**a**) Micropore region. (**b**) Mesoporous region.

Figure 5 shows the N_2_ adsorption-desorption isotherm of the composite molecular sieves prepared by single and secondary crystallization methods.

**Figure 6:**
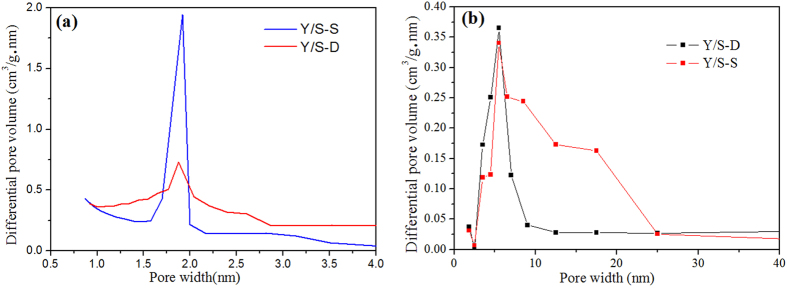
Distribution curves of Y/S-S and Y/S-D pore sizes.

The N_2_ adsorption-desorption isotherms of the Y/SBA-15 composite molecular sieves prepared by the two methods showed obvious microporous and mesoporous characteristics, which demonstrated that they were composite molecular sieves with composite pore structures. The saturated adsorption capacity of Y/S-D was around 175 cm^3^/g, while that of Y/S-S was around 140 cm^3^/g. Due to the irregular growth, there was a certain amount of defect in the open-framework structure of the molecular sieves prepared by single crystallization, resulting in lower specific surface area and pore volume (the detailed properties are given in Table 1). This was verified by the appearance of the relatively wide hysteresis loop between the adsorption and desorption bands on the Y/S-S isotherm. The distribution of pore sizes is shown in Fig. 6.

From Fig. 6, it can be seen that the pore size distribution of the composite molecular sieves via single crystallization was relatively wide, while the pore size distribution of the composite molecular sieves via secondary crystallization was relatively narrow. This indicates that the micropores and mesopores of the composite molecular sieves formed via secondary crystallization were more regular and ordered, and with similar pore sizes. This difference in the pore size distributions between the two composite molecular sieves is likely due to the limitations of the single crystallization method on the crystal growth orientation of the molecular sieves, resulting in irregular mesoporous channel orientation. On the other hand, the secondary crystallization method promoted regular and ordered growth of mesoporous phase on the surface of the microporous phase^14^.

The crystallization curve and the crystal structure parameters of the composite molecular sieves were obtained to further investigate the crystal growth mechanism of the Y/S-D core-shell composite molecular sieves.

**Figure 7:**
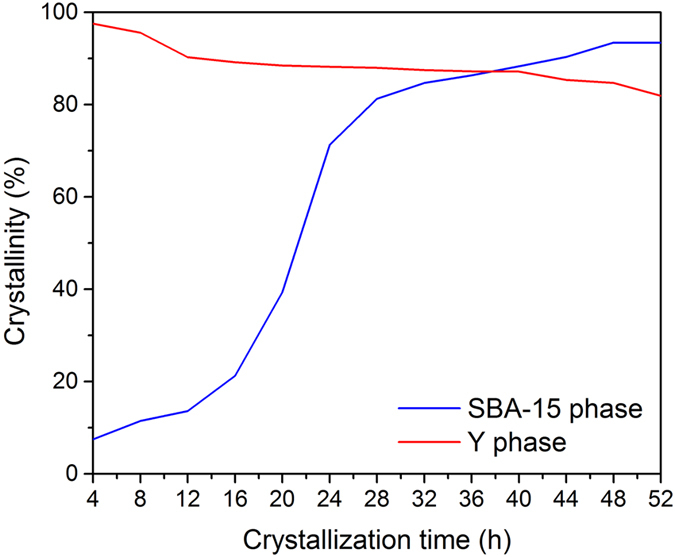
Crystallization curves of the core phase and the shell phase of Y/S-Dcomposite molecular sieves.

Figure 7 shows the crystallization curves of each phase of the composite molecular sieves prepared by secondary crystallization method. It can be seen from Fig. 7 that at the low temperature stage, the relative crystallinity of the SBA-15 phase increased slowly. This is due to the relatively high supersaturation degree of the initial gel, which promotes the nucleation and initial growth of the shell phase SBA-15 on the outer surface of the core phase Y. In addition, low temperature treatment favored the contact between the SBA-15 primary building units and Y, thus increasing the interaction and possibility of combination between the two phases. The high temperature stage provided a good platform for the crystal growth of the shell phase molecular sieves, leading to a more uniform crystal growth on the primary building units formed at the low temperature stage^17^. In the case of single crystallization treatment, complex crystals of the composite molecular sieves were formed in large amounts. This is likely due to the fact that at high temperature, the SBA-15 initial gel would rapidly go through the nucleation and crystal growth processes, leading to unstable contact of the formed nucleus and Y molecular sieve surface or monotonic growth orientation. Thus, the composite molecular sieve crystals prepared by single crystallization method were irregular.

### NH_3_-TPD analysis of the two composite molecular sieves

**Figure 8:**
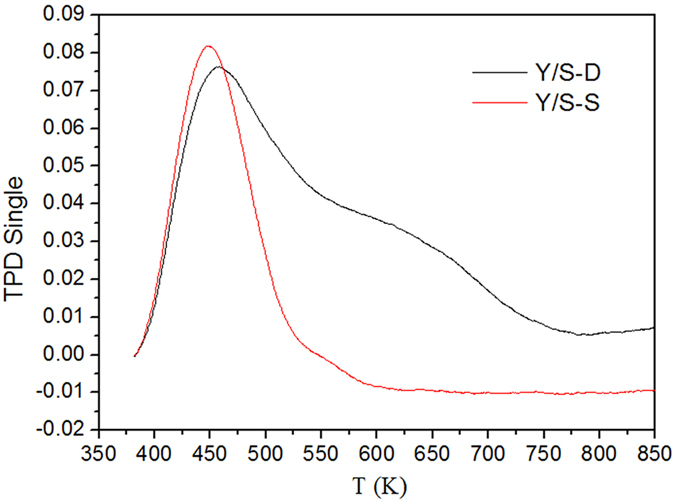
The NH_3_-TPD spectra of Y/S-S and Y/S-D.

NH_3_-TPD was used to study the distribution and intensities of the surface acid sites of the YS-S and YS-D. The results are shown in Fig. 8.

As shown in Fig. 8, both the composite molecular sieves had NH_3_ desorption peaks at around 400~500 K and 550~700 K. The NH_3_ desorption peak of Y/S-D was significantly stronger than that of Y/S-S, indicating that the acidity of Y/S-D was stronger than that of Y/S-S. Thus, the total acidity of the composite molecular sieves via secondary crystallization was higher than that of the composite molecular sieves via single crystallization. If the catalyst is too weakly acidic, pyrolysis and oligomerization of *n*-pentane cannot be completed. If the acidity of the catalyst is too strong, it would lead to over-pyrolysis of *n*-pentane, reducing the yield of the desired aromatic products. Due to their appropriate acidity, the composite molecular sieves prepared via secondary crystallization could ensure the high reactivity and selectivity of the *n*-pentane aromatization reaction^10^,^14^.

### Evaluation of aromatization reaction

**Figure 9:**
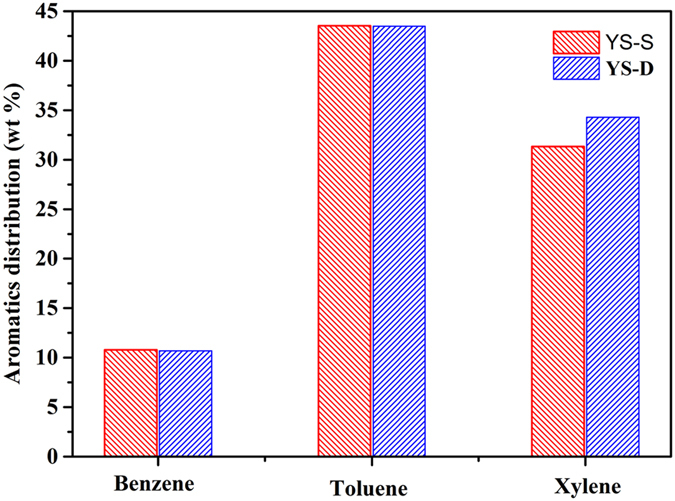
Aromatization performance of the two composite molecular sieve samples.

The results of *n*-pentane aromatization reactions catalyzed by different composite molecular sieves are shown in Fig. 9. As seen in Fig. 9, when composite zeolite Y/S-S (via single crystallization) was used as the catalyst, the yields of benzene and toluene were higher compared to using core-shell composite Y/S-D (via secondary crystallization). However, Y/S-D offered higher yield of xylenes, which have higher application value. In addition, the conversion obtained by using Y/S-D was 96.6% which was higher than those obtained by using Y/S-S (94.7%). This might be because the SBA-15 mesoporous phase of Y/S-D is uniformly distributed on the outer surface of the Y core. As a result, there is a relatively strong acid environment (provided by the microporous molecular sieve Y) suitable for aromatization reaction, as well as ordered and connected channels with a relatively large mesoporous space structure (provided by mesoporous molecular sieve SBA-15). The latter property favors the entrance of the sterically hindered *n*-pentane molecules, and facilitates shape selectivity and the exit of large products such as benzene, toluene and xylenes. Thus, the composite molecular sieve prepared by secondary crystallization provided more appropriate acidity and more regular mesoporous channel structure, resulting in better catalytic performance in the aromatization reaction.

## Conclusions

Two Y/SBA-15 composite molecular sieves were prepared by a facile method, and the effects of key crystallization conditions on the degree of crystallization and the open-framework structure of the composite molecular sieves were studied. The results indicated that the desired core-shell composite molecular sieves were obtained at the crystallization time of 36 hours, crystallization temperature of 100 °C and by secondary crystallization. Under these conditions, the mesoporous molecular sieve shell could be effectively coated on to the outer surface of the microporous molecular sieve nucleus. The open-framework structure was more regular and the crystal morphology was further improved when secondary crystallization was employed for preparation of the composite molecular sieves. The acidity of the core-shell composite molecular sieves and their open-framework structure showed good correlation, resulting in excellent catalytic performance in the aromatization reaction.

## Additional Information

**How to cite this article**: Shi, C.-W. *et al.* Investigation of Y/SBA Composite Molecular sieves Morphology Control and Catalytic Performance for *n*-Pentane Aromatization. *Sci. Rep.* **6**, 23826; doi: 10.1038/srep23826 (2016).

## Figures and Tables

**Table 1:** Selected crystal structural properties of the two samples.

Sample	*S*_*BET*_/(m^2^·g^−1^)	*V*_*BJHDesorption*_/(cm^3^·g^−1^)	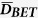 /nm	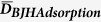 /nm	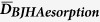 /nm
YS-S	406.5881	0.3255	4.0501	5.7397	5.3625
YS-D	493.1487	0.2466	2.8835	1.7858	1.7276
